# Supplementary material for: Whole-soil warming leads to substantial soil carbon emission in an alpine grassland
Source: Nat Commun. 2024 May 27;15:4489. doi: 10.1038/s41467-024-48736-w (PMC11130387; doi:10.1038/s41467-024-48736-w)
Supplement: Supplementary file 1 — Supplementary Information [file 41467_2024_48736_MOESM1_ESM.pdf]

Supplementary materials

Whole-soil warming leads to substantial soil carbon emission in an alpine grassland

Ying Chen[1], Wenkuan Qin[1], Qiufang Zhang[1], Xudong Wang[1], Jiguang Feng[1], Mengguang Han[1], Yanhui Hou[1], Hongyang Zhao[1], Zhenhua Zhang[2], Jin-Sheng He[1,3], Margaret S. Torn[4,5], Biao Zhu[1]*

[1]Institute of Ecology and Ministry of Education Key Laboratory for Earth Surface Processes, College of Urban and Environmental Sciences, Peking University, Beijing 100871, China.

[2]Qinghai Haibei National Field Research Station of Alpine Grassland Ecosystem and Key Laboratory of Adaptation and Evolution of Plateau Biota, Northwest Institute of Plateau Biology, Chinese Academy of Sciences, Xining 810008, China.

[3]State Key Laboratory of Herbage Improvement and Grassland Agro-ecosystems and College of Pastoral Agricultural Science and Technology, Lanzhou University, Lanzhou 730000, China.

[4]Climate and Ecosystem Sciences, Lawrence Berkeley National Laboratory, Berkeley, CA 94720, USA.

[5]Energy and Resources Group, University of California, Berkeley, Berkeley, CA 94720, USA.

*Corresponding author:
E-mail: biaozhu@pku.edu.cn

Supplementary Figure 1. **Photos of control and warming plots. a** Aerial photo of the study platform. **b** Photo of a warming plot.

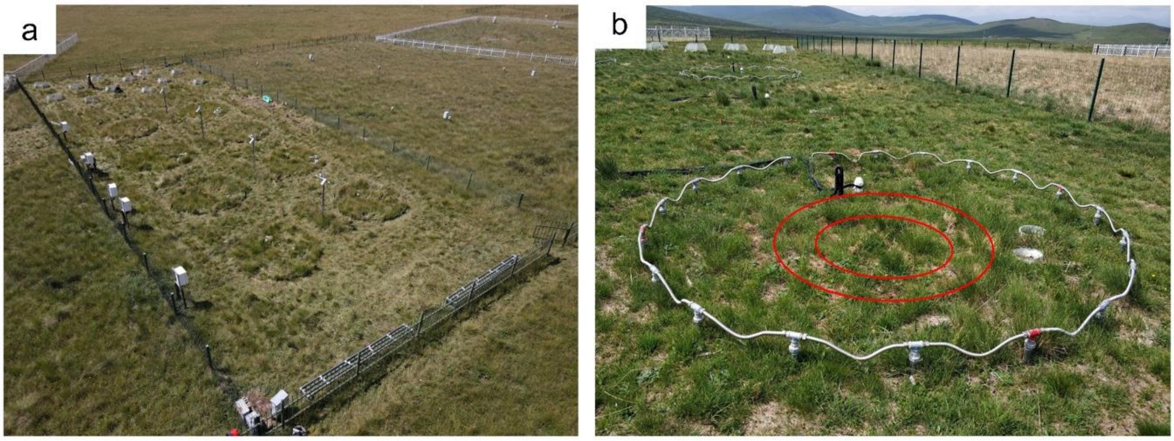

Supplementary Figure 2. **Temporal patterns in daily mean (± se, *n* = 4, biologically independent samples) <u>soil temperature</u> across the soil profile (5, 10, 20, 30, 40, 60, 80, and 100 cm) in control and warming treatments continuously over four years (from June 2018 to September 2021).** Different colors mean different treatments (blue indicates control and red indicates warming). Source data are provided as a Source Data file.

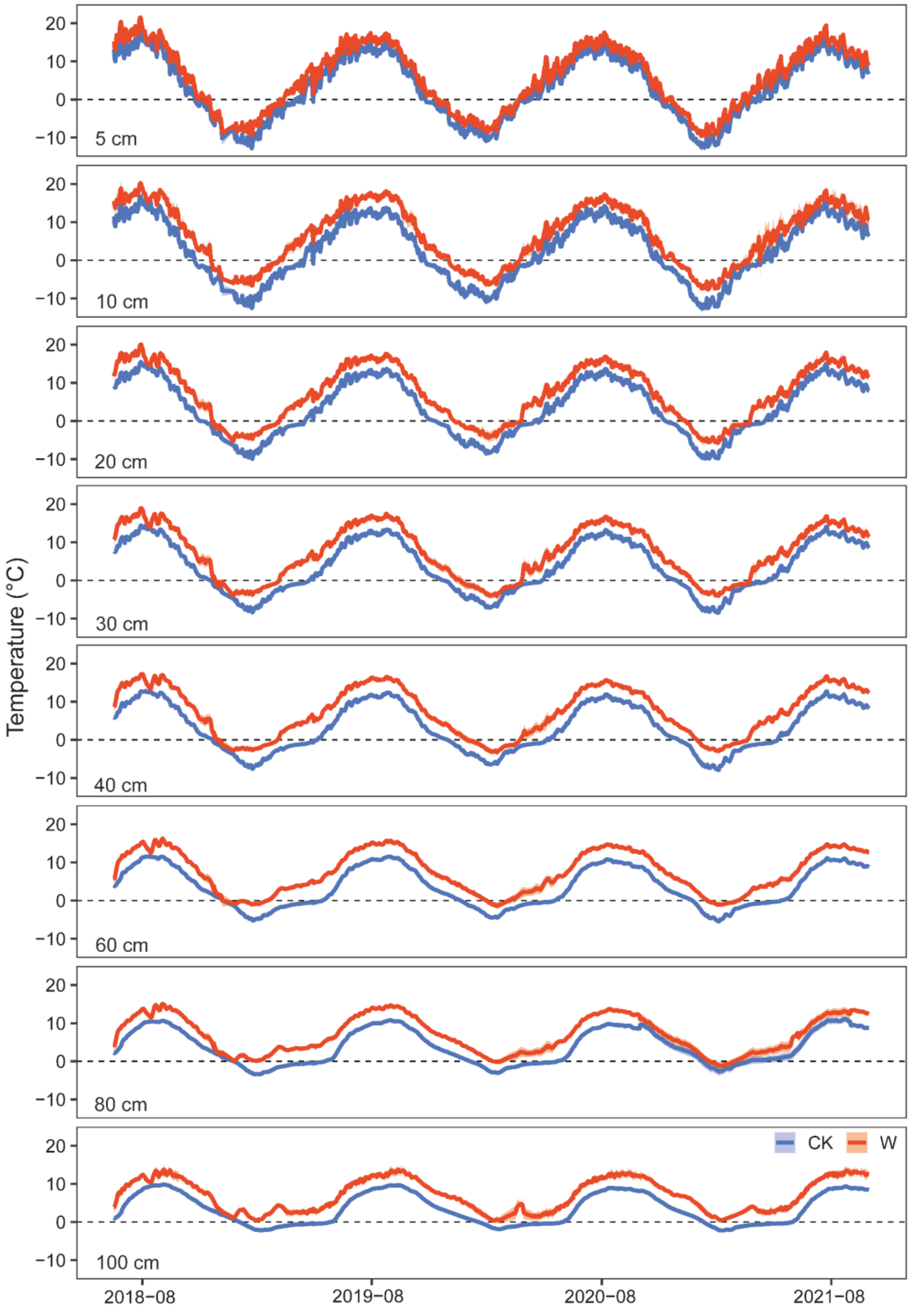

Supplementary Figure 3. **Temporal patterns in daily mean (± se, *n* = 4, biologically independent samples) <u>soil moisture</u> across the soil profile (10, 20, 30, 40, 60, and 80 cm) in control and warming treatments continuously over four years (from June 2018 to September 2021).** Different colors mean different treatments (blue indicates control and red indicates warming). Source data are provided as a Source Data file.

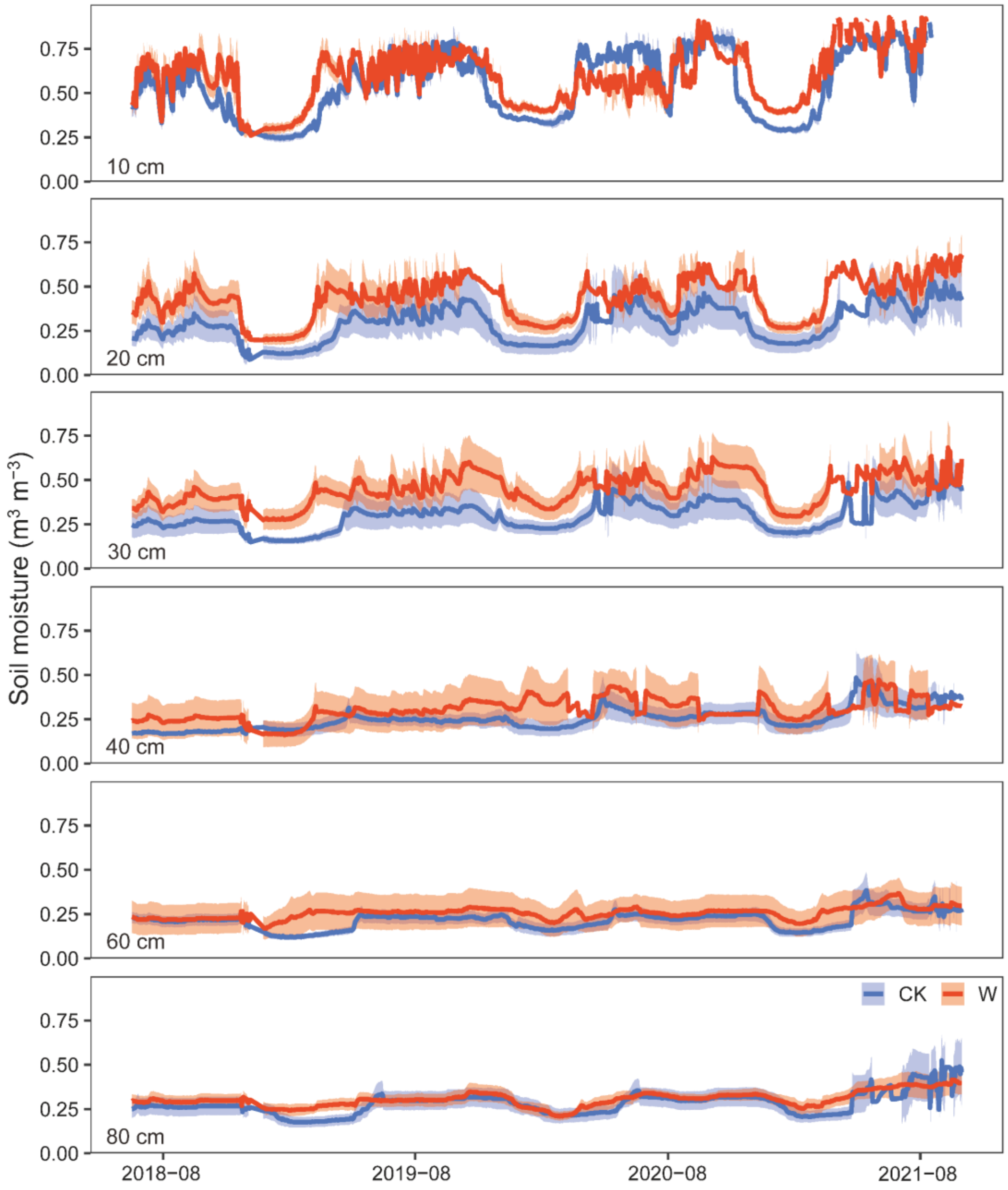

Supplementary Figure 4. **The temporal dynamics of plant properties to warming over four years (2018-2021). a** ANPP (aboveground net primary productivity). **b** AGB (aboveground biomass). **c** BNPP (belowground net primary productivity). **d** BGB (belowground biomass). The repeated measures ANOVA was used to test the effects of warming treatment and year on plant properties and then the Bonferroni test was done to assess the effect of warming over time. Different colors mean different treatments (blue indicates control and red indicates warming). Bars are mean ± standard errors (n = 4, biologically independent samples). Statistical significance and the difference between control and warming treatment are shown by asterisks († $P < 0.10$, * $P < 0.05$, ** $P < 0.01$, *** $P < 0.001$). Source data are provided as a Source Data file.

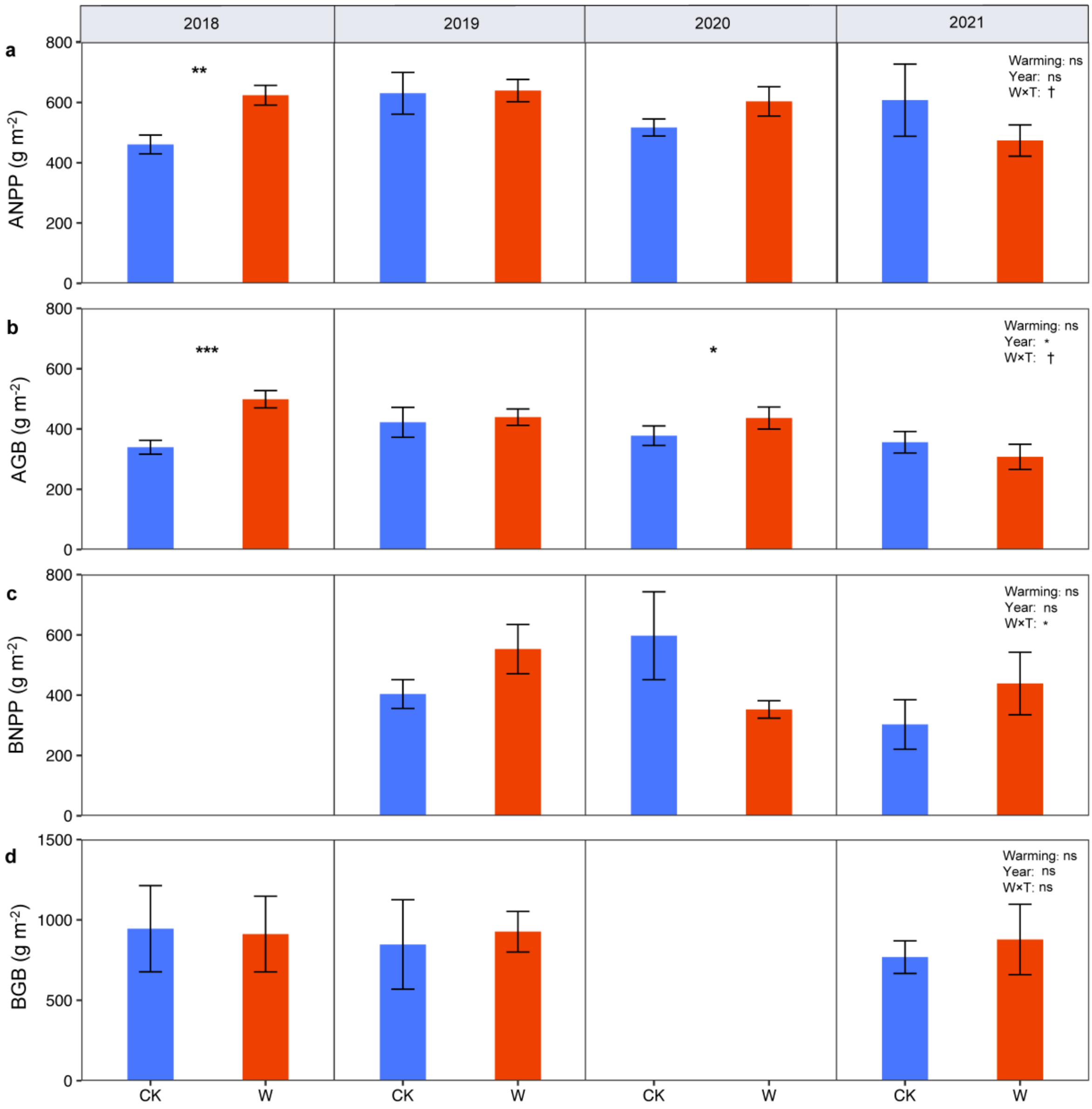

Supplementary Figure 5. **The response of plant properties to warming averaged over four years (2018-2021). a** ANPP (aboveground net primary productivity). **b** AGB (aboveground biomass). **c** BNPP (belowground net primary productivity). **d** BGB (belowground biomass). Different colors mean different treatments (blue indicates control and red indicates warming). A paired *t*-test (two-sided) was used to determine the effects of warming treatments on mean plant properties. Bars are mean ± standard errors ($n = 4$, biologically independent samples). The warming effect is not significant ($P > 0.10$) for all plant properties. Source data are provided as a Source Data file.

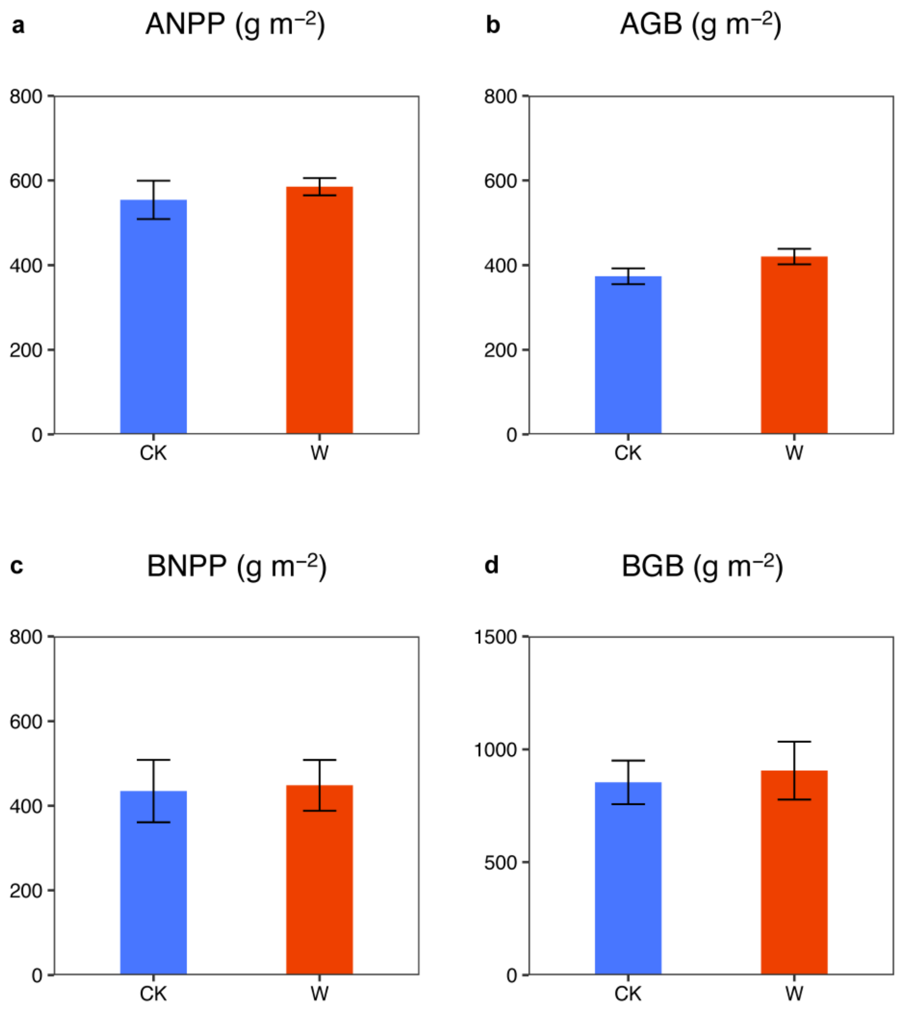

Supplementary Figure 6. **The response of soil properties to warming across the soil profile (0-100 cm) sampled in August 2019.** **a** SOC (soil organic carbon). **b** NH_4^+-N (ammonium nitrogen). **c** NO_3^--N (nitrate-nitrogen). **d** EOC (extractable organic carbon). **e** ETN (extractable total nitrogen). **f** MBC (microbial biomass carbon). **g** MBN (microbial biomass nitrogen). **h** BG (β-1,4-glucosidase). **i** NAG (β-1,4-N-acetyl-glucosaminidase). **j** AP (acid phosphatase). **k** CUE (carbon use efficiency), calculated based on microbial biomass and enzyme activity data using a stoichiometric method[1]. We use the linear mixed-effect models to estimate the effects of warming on soil properties across soil profile (0-100 cm) and a paired *t*-test (two-sided) was used to determine the effects of warming treatments on soil properties at different soil depths. Different colors mean different treatments (blue indicates control and red indicates warming). Bar values are the mean ± standard error ($n = 4$, biologically independent samples). Statistical significance is shown by asterisks († $P < 0.10$, * $P < 0.05$, ** $P < 0.01$, *** $P < 0.001$) or as non-significant (ns). The warming effect is not significant ($P > 0.10$) for most variables at all depths (0-10, 10-30, 30-60, and 60-100 cm). Source data are provided as a Source Data file.

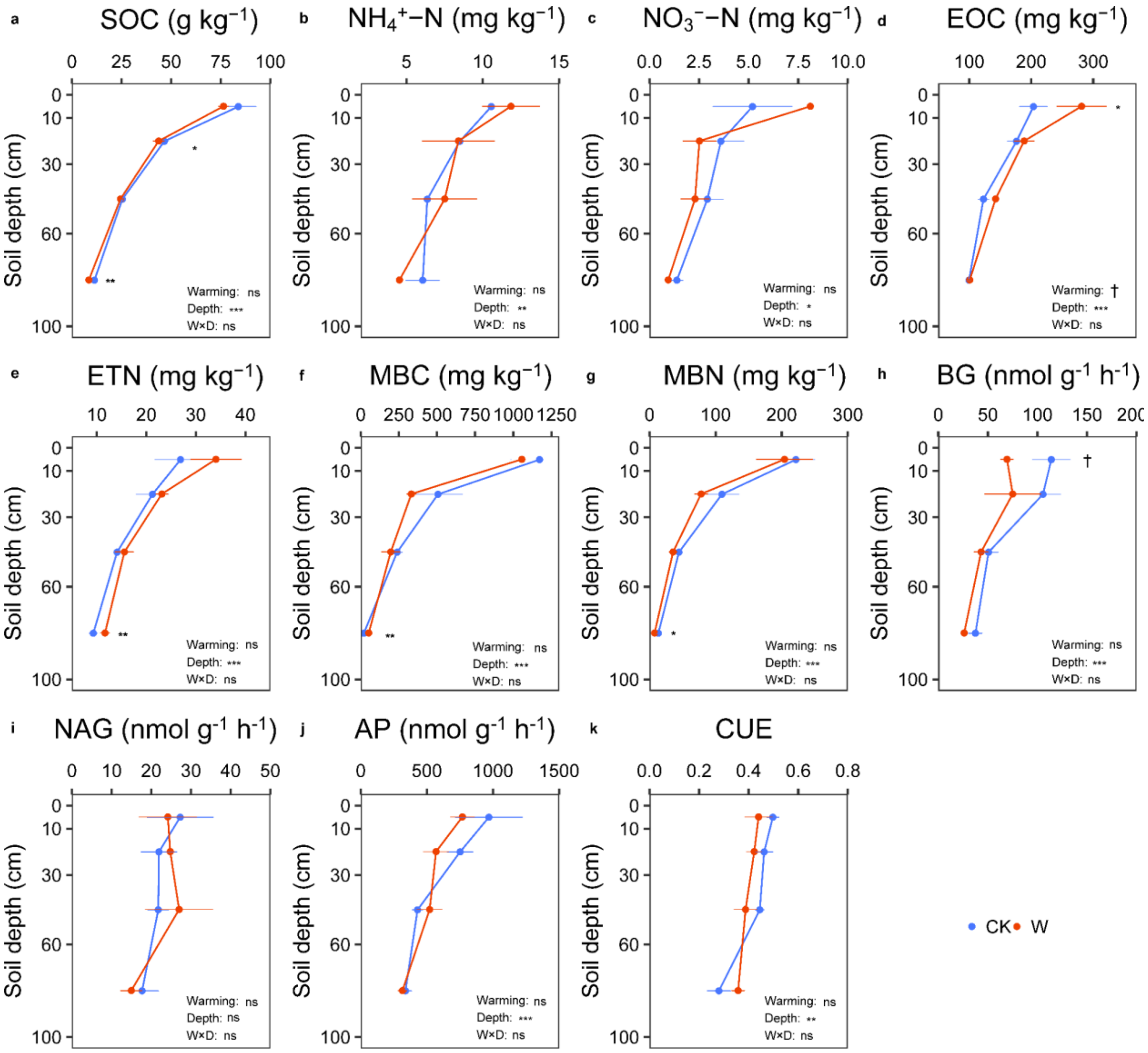
a
SOC (g kg^{-1})
0 25 50 75 100
Soil depth (cm)
0 10 30 60 100
Warming: ns
Depth: ***
W×D: ns
b
NH_4^+−N (mg kg^{-1})
5 10 15
Warming: ns
Depth: **
W×D: ns
c
NO_3^-−N (mg kg^{-1})
0.0 2.5 5.0 7.5 10.0
Warming: ns
Depth: *
W×D: ns
d
EOC (mg kg^{-1})
100 200 300
Warming: †
Depth: ***
W×D: ns
e
ETN (mg kg^{-1})
10 20 30 40
Warming: ns
Depth: ***
W×D: ns
f
MBC (mg kg^{-1})
0 250 500 750 1000 1250
Warming: ns
Depth: ***
W×D: ns
g
MBN (mg kg^{-1})
0 100 200 300
Warming: ns
Depth: ***
W×D: ns
h
BG (nmol g^{-1} h^{-1})
0 50 100 150 200
Warming: ns
Depth: ***
W×D: ns
i
NAG (nmol g^{-1} h^{-1})
0 10 20 30 40 50
Warming: ns
Depth: ns
W×D: ns
j
AP (nmol g^{-1} h^{-1})
0 500 1000 1500
Warming: ns
Depth: ***
W×D: ns
k
CUE
0.0 0.2 0.4 0.6 0.8
Warming: ns
Depth: **
W×D: ns
CK W

Supplementary Figure 7. **The response of soil properties to warming across the soil profile (0-100 cm) sampled in August 2021.** **a** SOC (soil organic carbon). **b** NH_4^+-N (ammonium nitrogen). **c** NO_3^--N (nitrate-nitrogen). **d** EOC (extractable organic carbon). **e** ETN (extractable total nitrogen). **f** MBC (microbial biomass carbon). **g** MBN (microbial biomass nitrogen). **h** BG (β-1,4-glucosidase). **i** NAG (β-1,4-N-acetyl-glucosaminidase). **j** AP (acid phosphatase). **k** CUE (carbon use efficiency), calculated based on microbial biomass and enzyme activity data using a stoichiometric method[1]. We use the linear mixed-effect models to estimate the effects of warming on soil properties across soil profile (0-100 cm) and a paired *t*-test (two-sided) was used to determine the effects of warming treatments on soil properties at different soil depths. Different colors mean different treatments (blue indicates control and red indicates warming). Bar values are the mean ± standard error ($n = 4$, biologically independent samples). Statistical significance is shown by asterisks († $P < 0.10$, * $P < 0.05$, ** $P < 0.01$, *** $P < 0.001$) or as non-significant (ns). The warming effect is not significant ($P > 0.10$) for most variables at all depths (0-10, 10-30, 30-60, and 60-100 cm). Source data are provided as a Source Data file.

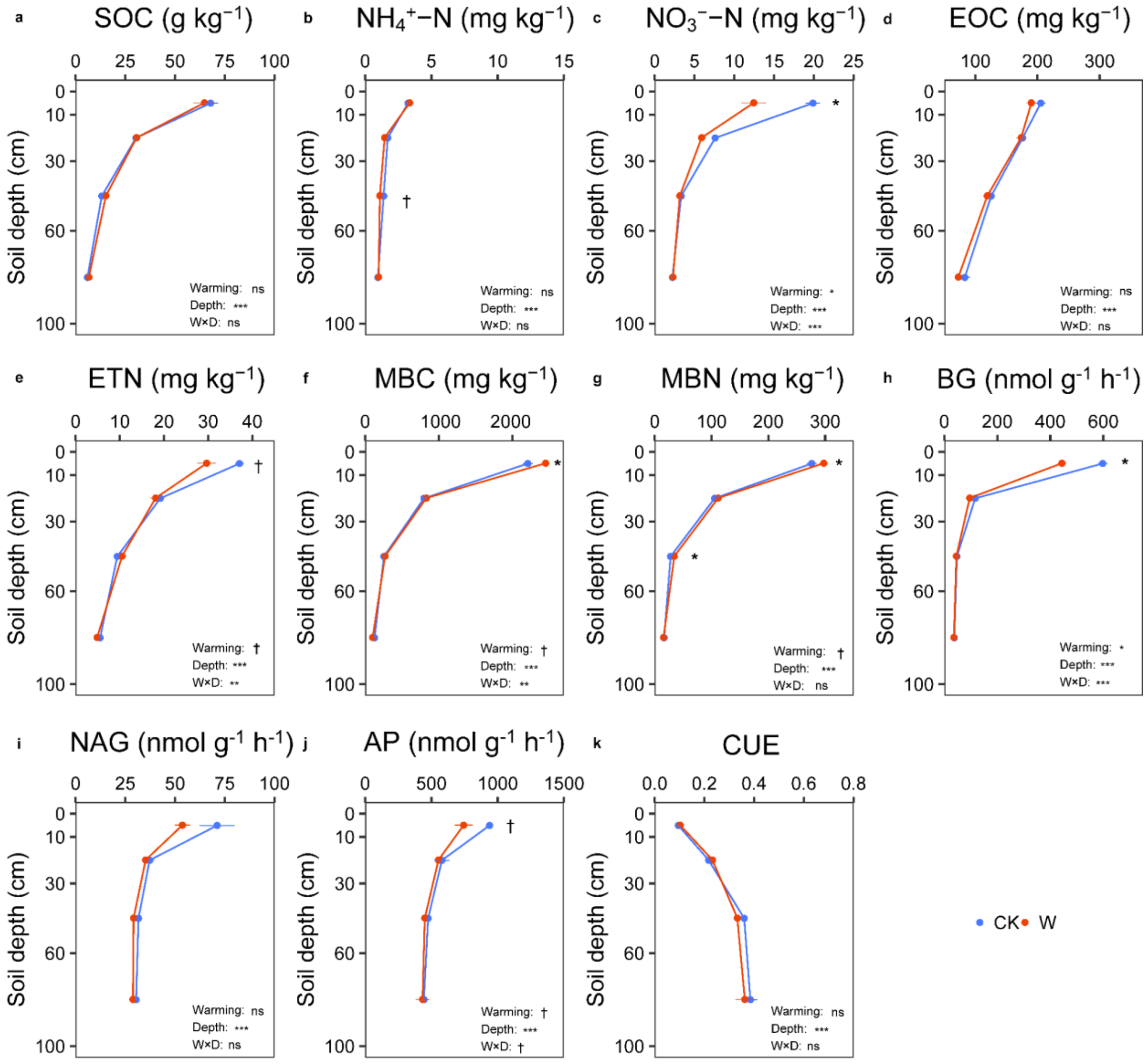
a SOC (g kg^{-1})
b NH_4^+−N (mg kg^{-1})
c NO_3^-−N (mg kg^{-1})
d EOC (mg kg^{-1})
e ETN (mg kg^{-1})
f MBC (mg kg^{-1})
g MBN (mg kg^{-1})
h BG (nmol g^{-1} h^{-1})
i NAG (nmol g^{-1} h^{-1})
j AP (nmol g^{-1} h^{-1})
k CUE
Soil depth (cm)
Warming: ns
Depth: ***
W×D: ns
Warming: ns
Depth: ***
W×D: ns
Warming: *
Depth: ***
W×D: ***
Warming: ns
Depth: ***
W×D: ns
Warming: †
Depth: ***
W×D: **
Warming: †
Depth: ***
W×D: **
Warming: †
Depth: ***
W×D: ns
Warming: *
Depth: ***
W×D: ***
Warming: ns
Depth: ***
W×D: ns
Warming: †
Depth: ***
W×D: †
Warming: ns
Depth: ***
W×D: ns
CK
W

Supplementary Figure 8. **The mean soil CO_2 efflux partitioned into SOC-derived (heterotrophic respiration) and root-derived (autotrophic respiration) components for growing season measurements. a** 2018. **b** 2019. **c** 2020. **d** 2021. The repeated measures ANOVA was used to test the effects of warming treatment and year on soil CO_2 efflux and then the Bonferroni test was done to assess the effect of warming over time. Different colors mean different treatments (blue indicates control and red indicates warming). Statistical significance and the difference between control and warming treatment are shown by asterisks († $P < 0.10$, * $P < 0.05$, ** $P < 0.01$, *** $P < 0.001$, $n = 4$, biologically independent samples) or as non-significant (ns). Boxes represent the interquartile range (IQR), and whiskers indicate the furthest point within 1.5 × IQR above or below the IQR. Values beyond this range are plotted as individual points. The central line indicates the median. Source data are provided as a Source Data file.

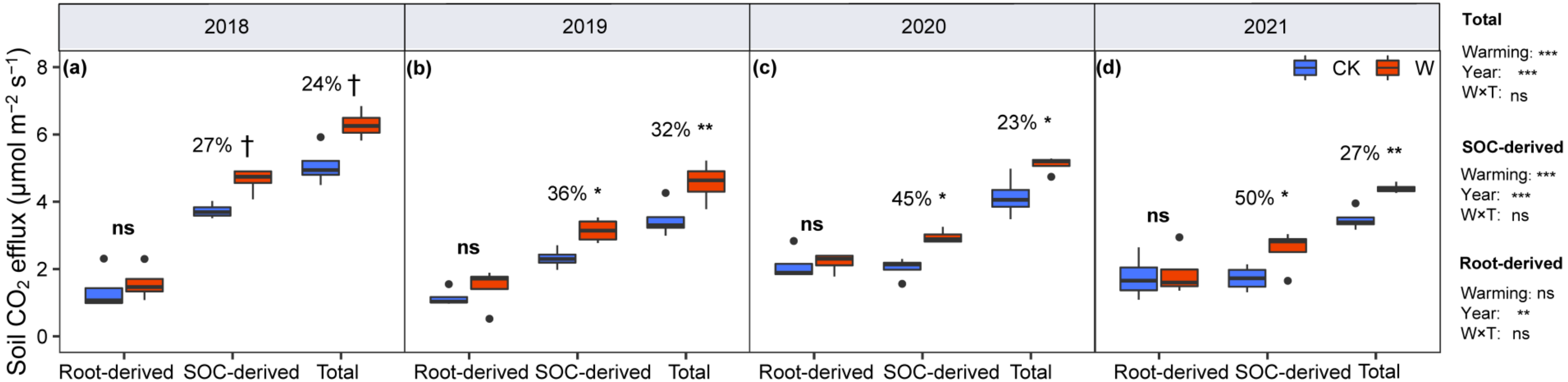

Supplementary Figure 9. **The mean soil CO_2 efflux (corrected value) partitioned into SOC-derived (heterotrophic respiration) and root-derived (autotrophic respiration) components for growing season measurements in four years of warming periods (from June 2018 to September 2021).** The soil CO_2 efflux was corrected by the "*SpATS*" R package to reduce spatial heterogeneity (please see the text for details). Different colors mean different treatments (blue indicates control and red indicates warming). A paired *t*-test (two-sided) was used to determine the effects of warming treatments on mean soil CO_2 efflux. Differences between control and warming treatment is shown by asterisks († $P < 0.10$, * $P < 0.05$, ** $P < 0.01$, *** $P < 0.001$, $n = 4$, biologically independent samples) or as non-significant (ns). Boxes represent the interquartile range (IQR), and whiskers indicate the furthest point within 1.5 × IQR above or below the IQR. Values beyond this range are plotted as individual points. The central line indicates the median. Source data are provided as a Source Data file.

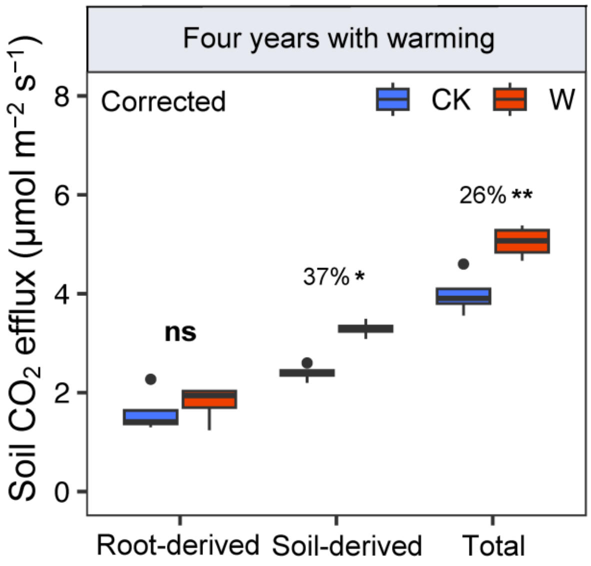

Supplementary Figure 10. **Contribution of SOC-derived and root-derived sources to total CO_2 efflux over four years (from June 2018 to September 2021). a** SOC-derived source (heterotrophic respiration). **b** Root-derived source (autotrophic respiration). The repeated measures ANOVA was used to test the effects of warming treatment and time on contribution of SOC-derived and root-derived sources to total CO_2 efflux. In 2018-2020 growing seasons, measurements were made every two weeks; in 2021 growing season, measurements were made monthly. The light-blue shaded areas represent the pre-treatment period (before June 17, 2018), and the orange shaded areas represent four years of warming periods (from June 2018 to September 2021). Different colors mean different treatments (blue indicates control and red indicates warming). Bar values are the mean ± standard error (n = 4, biologically independent samples). Statistical significance is shown by asterisks († $P < 0.10$, * $P < 0.05$, ** $P < 0.01$, *** $P < 0.001$) or as non-significant (ns). Source data are provided as a Source Data file.

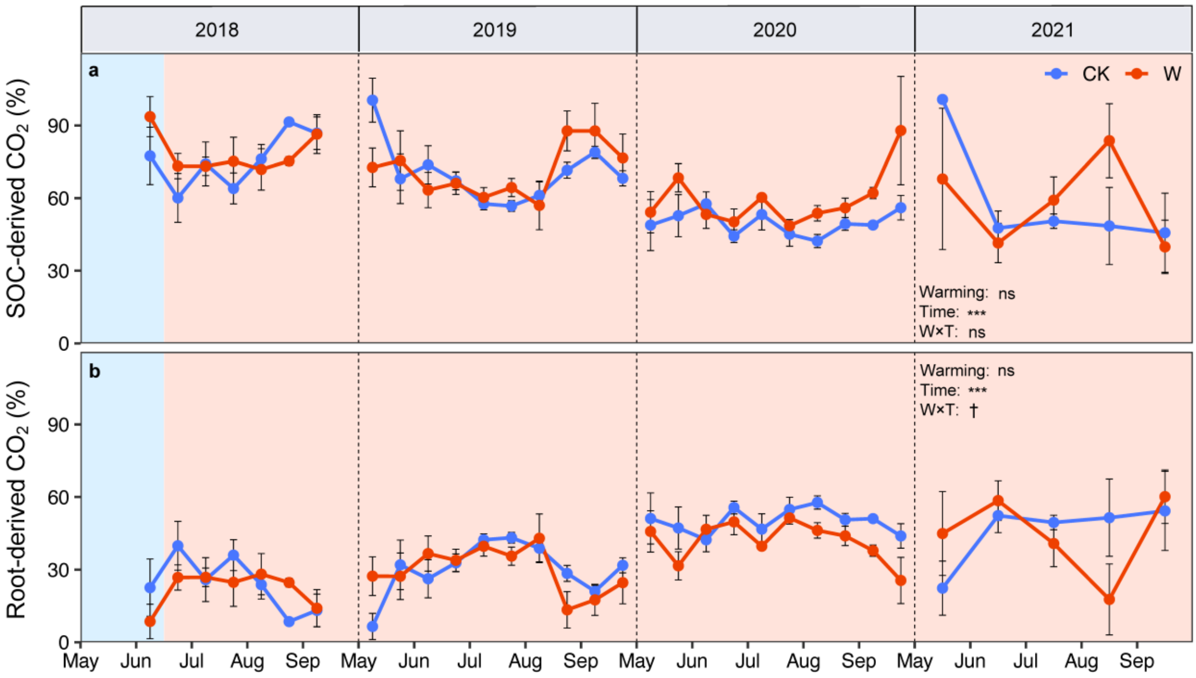

Supplementary Figure 11. **The average contribution of SOC-derived and root-derived sources to total CO_2 efflux for growing season measurements over four years of warming periods (from June 2018 to September 2021).** Different colors mean different treatments (blue indicates control and red indicates warming). A paired *t*-test (two-sided) was used to determine the effects of warming treatments on average contribution of SOC-derived and root-derived sources to total CO_2 efflux.. Bars are mean ± standard errors (n = 4, biologically independent samples). The warming effect is not significant ($P > 0.10$) for both root-derived and SOC-derived sources. Source data are provided as a Source Data file.

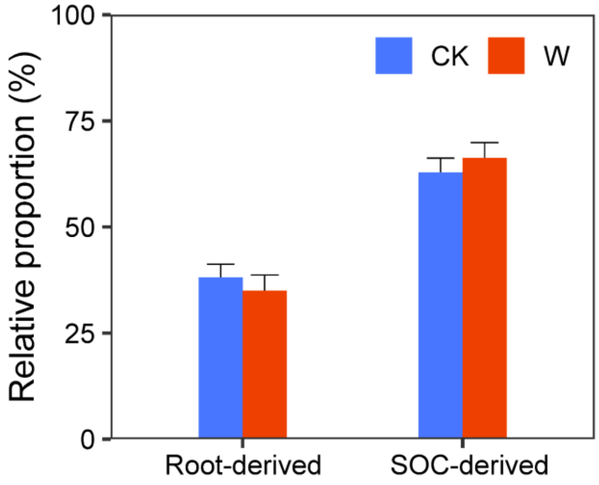

Supplementary Figure 12. **Effects of warming methods on soil respiration (Rs), heterotrophic respiration (Rh), and autotrophic respiration (Ra) in all grasslands globally (total data).** Circles and error bars represent average parameter estimates and 95% confidence intervals (CIs) in the linear mixed effects models (two–sided). IH: infrared heater, OTC: open top chamber. Other warming methods included heating cable, infrared reflector, greenhouse, and translocation. The Q_B statistical test was used to compare the differences in weighted effect sizes among groups divided by the warming method. A significant Q_B value ($P < 0.05$) suggested that the weighted effect sizes of a given variable differed among groups. The sample size (n) for each variable is in parentheses. Source data are provided as a Source Data file.

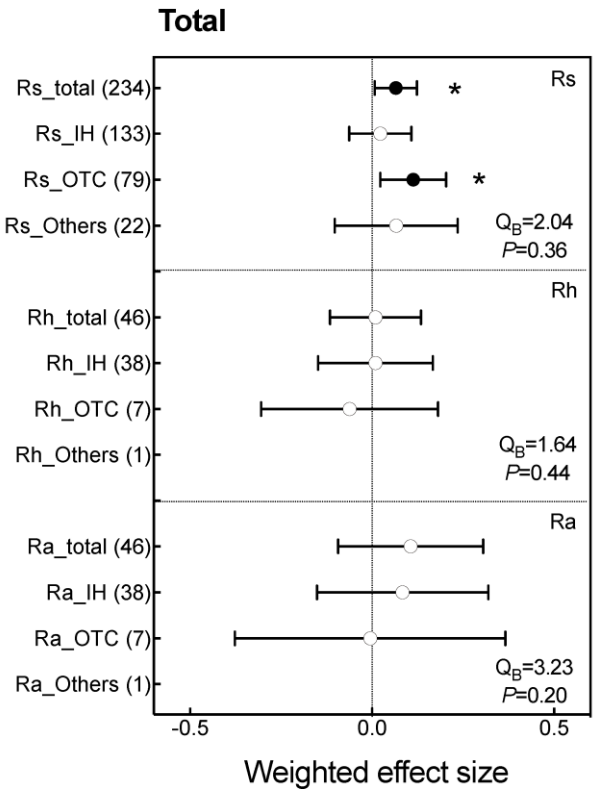

Supplementary Figure 13. **Model-averaged relative importance of the predictors of warming effects on soil respiration (total Rs, Rh, and Ra) in all grasslands globally. a-c,** total data; **d-f,** paired data. MAT, mean annual temperature; MAP, mean annual precipitation. Cut off (red dashed line) is set at 0.8 to differentiate between important and nonessential predictors. The importance is based on the sum of Akaike weights derived from the model selection using corrected Akaike's Information Criteria. Total data are from experiments that only measured total soil respiration (but did not separate it into heterotrophic and autotrophic components), while paired data are from experiments that separated total soil respiration into heterotrophic and autotrophic components. See Fig. 5 for the overall responses. Source data are provided as a Source Data file.

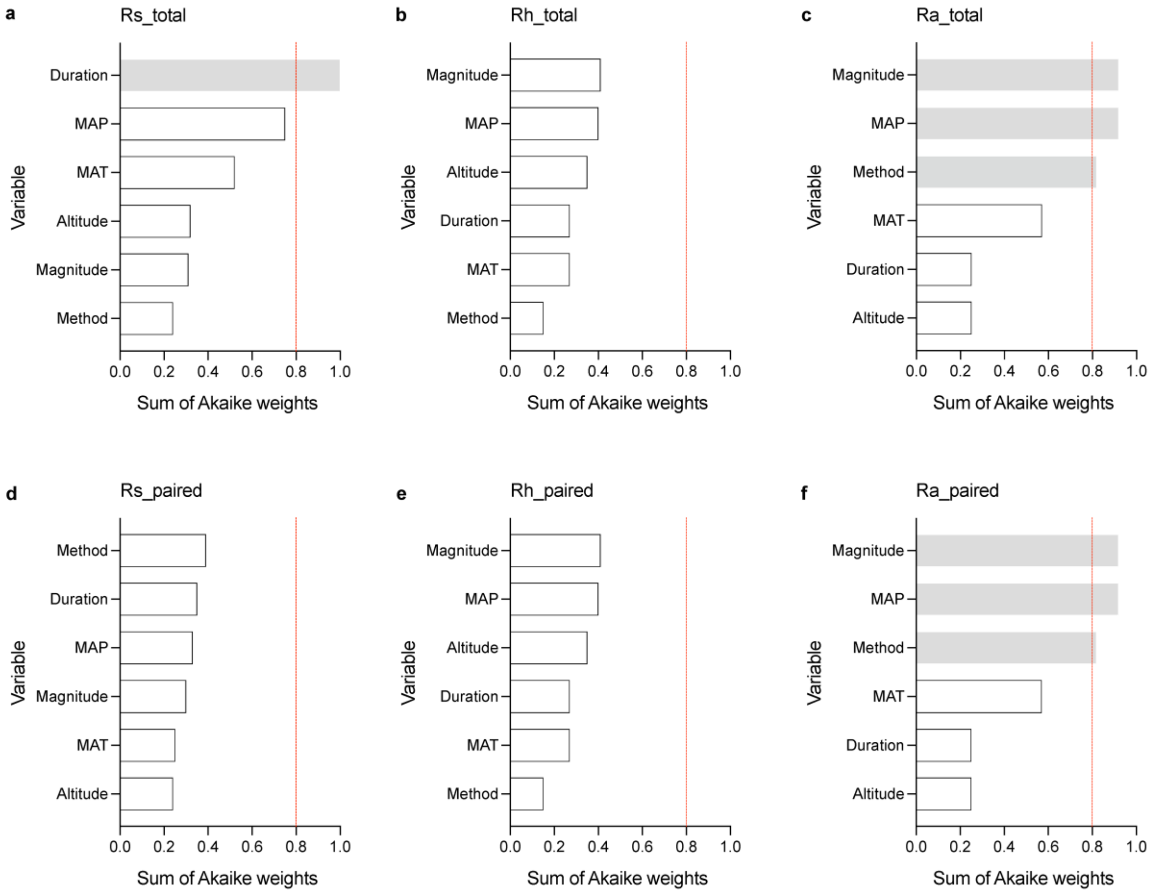

Supplementary Figure 14. **The relationships between the effect sizes of soil respiration (Rs) with the duration of the warming experiment (a, total data; b, paired data).** Total data are from experiments that only measured total soil respiration (but did not separate it into heterotrophic and autotrophic components), while paired data are from experiments that separated total soil respiration into heterotrophic and autotrophic components. Source data are provided as a Source Data file.

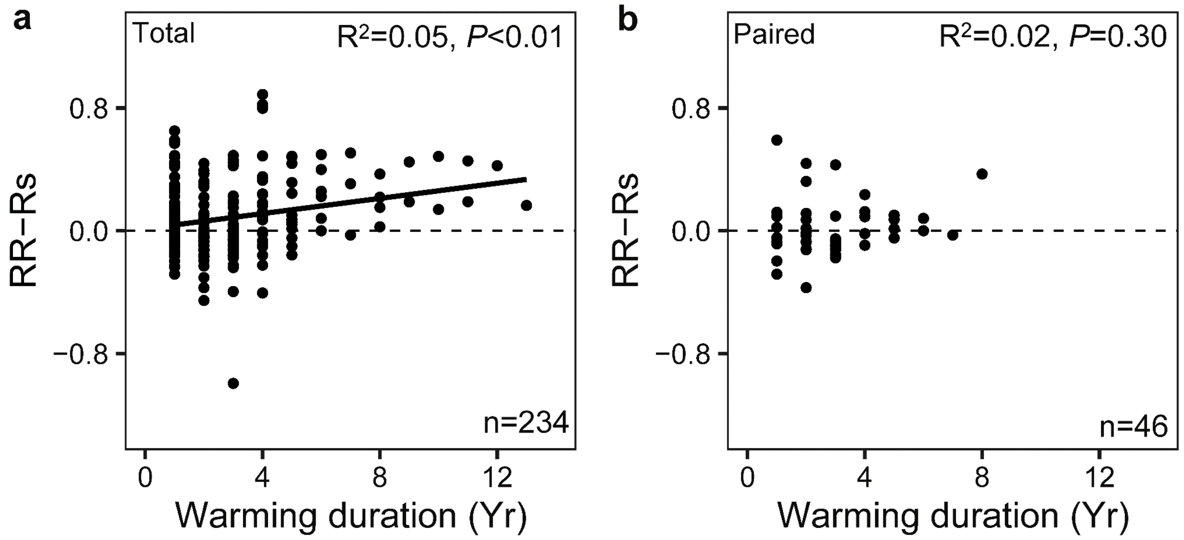

Supplementary Figure 15. **Distribution map of field warming experiments that measured soil CO_2 efflux across all grasslands globally (47 sites, 59 experiments, 72 papers).** Source data are provided as a Source Data file.

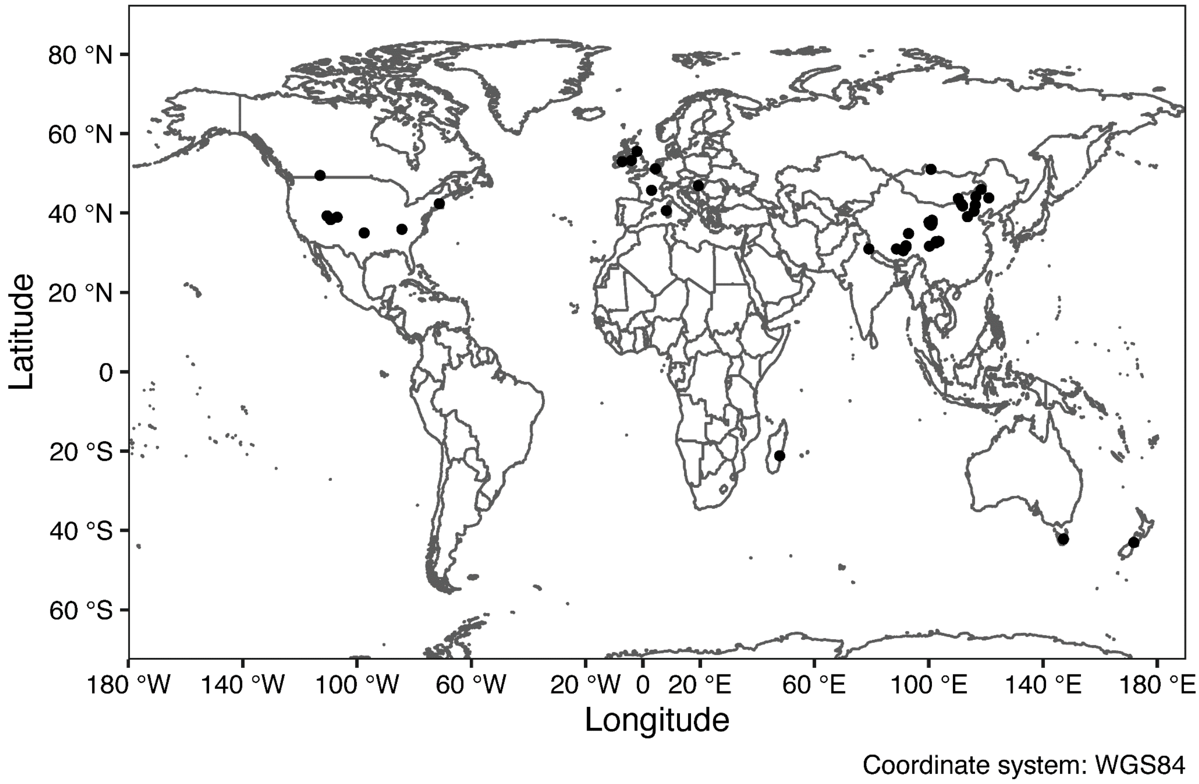

Supplementary Figure 16. **The number of observations included in this meta-analysis across all grasslands globally (234 observations, each observation means each total soil CO_2 flux (46 were also partitioned into SOC- and root-derived components) in paired control and warming treatment).** IH: infrared heater, OTC: open top chamber. Other warming methods included heating cable, infrared reflector, greenhouse, and translocation. Source data are provided as a Source Data file.

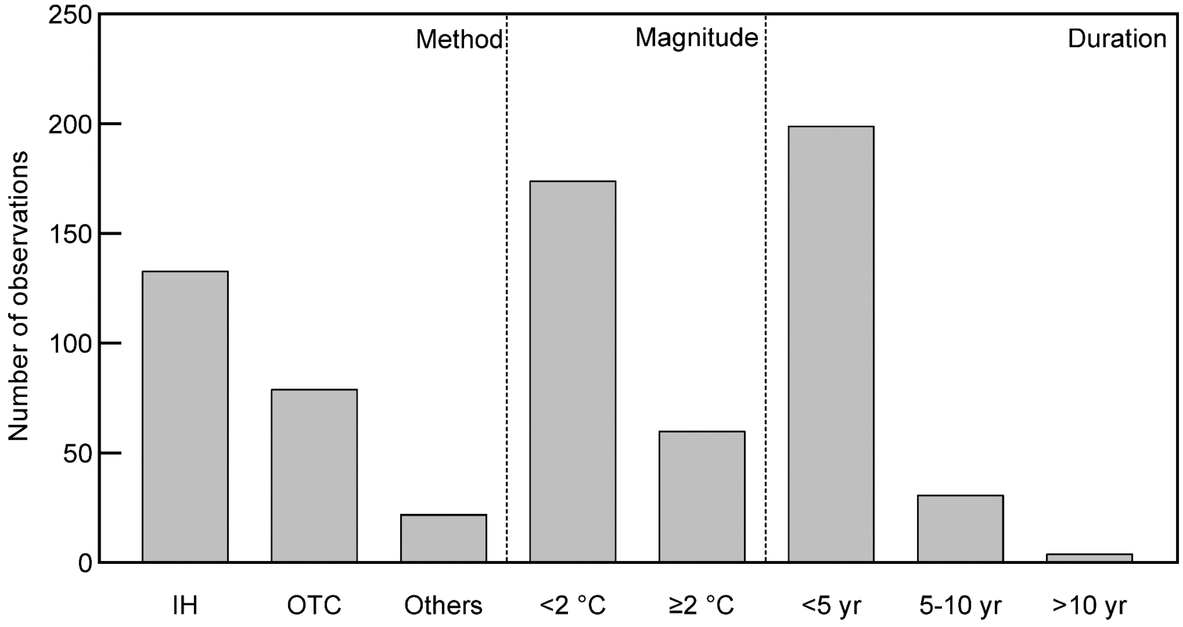

Supplementary Figure 17. **PRISMA Flow Diagram showing the study selection process in the meta-analysis.**

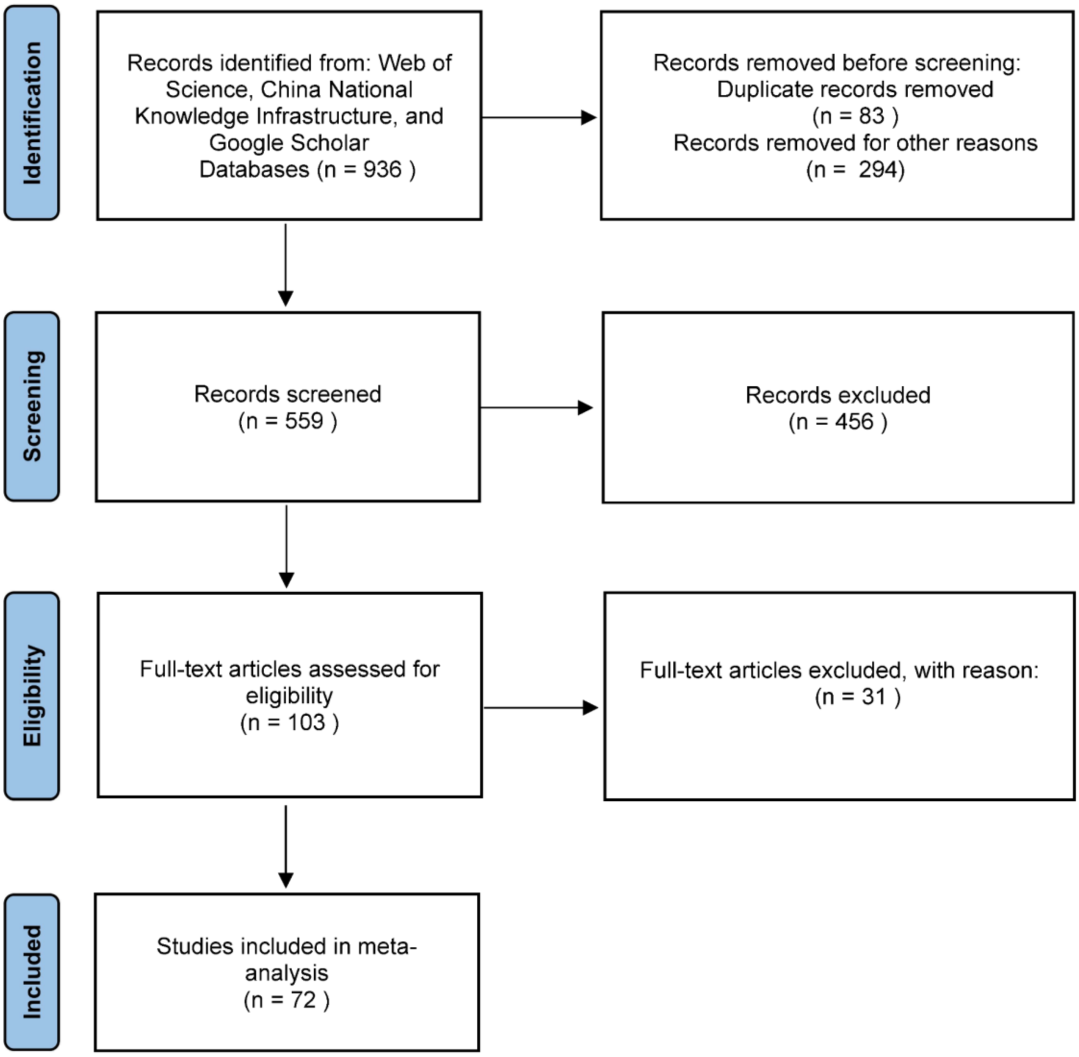

Supplementary Table 1. **Comparison of mean soil CO_2 efflux (total, SOC-derived, and root-derived) from this study with two other whole-soil warming experiments (in forests) and one whole-ecosystem warming experiment (in a peatland) across the world.**

Study	Warming type	Location	Ecosystem	Warming magnitude (°C)	Warming duration (yr)	Soil Profile heated （cm）	Warming effect on soil CO_2 efflux (in % increase)		
							Total	SOC-derived	Root-derived
**This study**	**Whole-soil**	**Qinghai, China**	**Alpine grassland**	**4.00**	**4**	**0-100**	**26**	**37**	**12**
Pries et al. 2017[2]	Whole-soil	California, US	Temperate forest	4.00	2	0-100	34	——	——
Soong et al. 2021[3]	Whole-soil	California, US	Temperate forest	4.00	5	0-100	30	——	——
Nottingham et al. 2020[1]	Whole-soil	Barro Colorado, Panama	Tropical forest	4.00	2	0-120	55	68	32
Hanson et al. 2020[4]	Whole-ecosystem	Minnesota, US	Boreal peatland	2.25	3	0-300	——	27	——
Hanson et al. 2020[4]	Whole-ecosystem	Minnesota, US	Boreal peatland	4.50	3	0-300	——	24	——
Hanson et al. 2020[4]	Whole-ecosystem	Minnesota, US	Boreal peatland	6.75	3	0-300	——	48	——
Hanson et al. 2020[4]	Whole-ecosystem	Minnesota, US	Boreal peatland	9.00	3	0-300	——	45	——

Supplementary Table 2. **Initial pre-treatment plant and soil properties across the soil profile (0-10, 10-30, 30-50, 50-100 cm) in the alpine grassland.** BGB, belowground biomass; SOC, soil organic carbon; TN, total nitrogen; EOC, extractable organic carbon; ETN, extractable total nitrogen; NH_4^+-N, ammonium nitrogen; NO_3^--N, nitrate-nitrogen; AN, available nitrogen (ammonium plus nitrate). Values are pre-treatment and were determined in August 2017 before the warming experiment was started (mean ± standard error, *n* = 4).

Depth	0-10 cm	10-30 cm	30-50 cm	50-100 cm
BGB (g m^{-2})	1376.2±384.0	112.3±31.4	126.1±55.2	34.1±19.9
pH	7.63±0.13	8.10±0.02	8.38±0.13	8.64±0.02
SOC (g kg^{-1})	72.93±17.59	37.12±0.85	24.27±1.23	23.43±12.21
TN (g kg^{-1})	7.18±1.64	4.28±0.09	2.96±0.14	2.48±1.02
EOC (mg kg^{-1})	433.6±38.3	356.9±11.0	279.9±23.3	168.7±9.2
ETN (mg kg^{-1})	62.74±6.76	33.24±1.32	24.44±3.72	8.73±0.62
NH_4^+-N (mg kg^{-1})	4.19±0.48	2.71±0.28	3.12±0.26	2.42±0.12
NO_3^--N (mg kg^{-1})	3.59±0.75	2.34±0.12	1.69±0.21	0.66±0.05
AN (mg kg^{-1})	7.78±0.92	5.05±0.83	4.81±1.38	3.07±0.28

Supplementary Table 3. **Detailed site information for studies (59 studies and 234 observations) included in our meta-analysis across all grasslands globally.** MAT, mean annual temperature (°C); MAP, mean annual precipitation (mm). Warming method: OTC, open top chamber; IH, infrared heater; others (heating cable, infrared reflector, greenhouse, and translocation). Response variables to warming include: soil respiration (Rs), soil heterotrophic respiration (Rh), and soil autotrophic respiration (Ra). The database and ariticles list of this meta-analysis were deposited at https://github.com/yancypku/soil-respiration.

**References**	**Altitude**	**MAT**	**MAP**	**Method**	**Duration**	**Magnitude**	**Response variables to warming**		
							**Rs**	**Rh**	**Ra**
Flanagan et al. 2013	951	5.2	396	OTC	1	0.85	√		
de Boeck et al. 2007	9	9.6	776	Others	1	3.00	√		
de Boeck et al. 2007	9	9.6	776	Others	2	3.00	√		
de Boeck et al. 2007	9	9.6	776	Others	1	3.00	√		
de Boeck et al. 2007	9	9.6	776	Others	2	3.00	√		
de Boeck et al. 2007	9	9.6	776	Others	1	3.00	√		
de Boeck et al. 2007	9	9.6	776	Others	2	3.00	√		
Li et al. 2013	345	16.2	905	IH	1	2.40	√	√	√
Li et al. 2013	345	16.2	905	IH	2	2.40	√	√	√
Li et al. 2013	345	16.2	905	IH	3	2.40	√	√	√
Wan et al. 2005	345	16.2	905	IH	1	2.40	√		
Luo et al. 2009	345	16.2	905	IH	2	2.40	√	√	√
Zhou et al. 2007	345	16.2	905	IH	3	1.10	√	√	√
Zhou et al. 2007	345	16.2	905	IH	4	1.40	√	√	√
Zhou et al. 2007	345	16.2	905	IH	5	1.20	√	√	√
Zhou et al. 2007	345	16.2	905	IH	6	1.60	√	√	√
de Dato et al. 2010	30	16.0	598	Others	1	0.10	√		

de Dato et al. 2010	30	16.0	598	Others	2	0.10	√		
de Dato et al. 2010	30	16.0	598	Others	3	0.10	√		
Gill 2014	1608	1.7	902	OTC	1	2.00	√		
Gill 2014	1608	1.7	902	OTC	2	2.00	√		
Gill 2014	1608	1.7	902	OTC	3	2.00	√		
Briones et al. 2009	309	8.0	900	Others	1	3.50	√		
Casella & Soussana 1997	850	8.6	759	Others	1	3.00	√		
Lu et al. 2013	4675	0.0	300	OTC	1	3.46	√		
Wan et al. 2009	1324	2.4	379	IH	1	1.71	√		
Wan et al. 2009	1324	2.4	379	IH	2	1.71	√		
Wan et al. 2009	1324	2.4	379	IH	3	1.71	√		
Wan et al. 2009	1324	2.4	379	IH	4	1.71	√		
Wan et al. 2009	1324	2.4	379	IH	1	0.32	√		
Wan et al. 2009	1324	2.4	379	IH	2	0.32	√		
Wan et al. 2009	1324	2.4	379	IH	3	0.32	√		
Wan et al. 2009	1324	2.4	379	IH	4	0.32	√		
Wan et al. 2009	1324	2.4	379	IH	1	0.42	√		
Wan et al. 2009	1324	2.4	379	IH	2	0.42	√		
Wan et al. 2009	1324	2.4	379	IH	3	0.42	√		
Wan et al. 2009	1324	2.4	379	IH	4	0.42	√		
Xia et al. 2009	1324	2.4	379	IH	1	1.79	√		
Xia et al. 2009	1324	2.4	379	IH	2	1.79	√		
Xia et al. 2009	1324	2.4	379	IH	3	1.79	√		
Xia et al. 2009	1324	2.4	379	IH	4	1.79	√		
Xia et al. 2009	1324	2.4	379	IH	5	1.79	√		
Wang et al. 2012	1456	3.4	248	IH	1	1.25	√		

Wang et al. 2012	1456	3.4	248	IH	2	1.25	√		
Wang et al. 2012	1456	3.4	248	IH	3	1.25	√		
Suseela & Dukes 2013	20	9.4	1130	IH	1	1.00	√		
Suseela & Dukes 2013	20	9.4	1130	IH	1	2.70	√		
Suseela & Dukes 2013	20	9.4	1130	IH	1	4.00	√		
Song et al. 2019	1324	2.4	379	IH	1	0.17	√		
Song et al. 2019	1324	2.4	379	IH	2	0.17	√		
Song et al. 2019	1324	2.4	379	IH	3	0.17	√		
Song et al. 2019	1324	2.4	379	IH	1	0.55	√		
Song et al. 2019	1324	2.4	379	IH	2	0.55	√		
Song et al. 2019	1324	2.4	379	IH	3	0.55	√		
Song et al. 2019	1324	2.4	379	IH	1	0.25	√		
Song et al. 2019	1324	2.4	379	IH	2	0.25	√		
Song et al. 2019	1324	2.4	379	IH	3	0.25	√		
Niu et al. 2009; Liu et al. 2009; Niu et al. 2011	1324	2.4	379	IH	1	1.17	√		
Niu et al. 2009; Liu et al. 2009; Niu et al. 2011	1324	2.4	379	IH	2	1.17	√		
Niu et al. 2009; Liu et al. 2009; Niu et al. 2011	1324	2.4	379	IH	3	1.17	√		
Niu et al. 2009; Liu et al. 2009; Niu et al. 2011	1324	2.4	379	IH	4	1.17	√		
Niu et al. 2009; Liu et al. 2009; Niu et al. 2011	1324	2.4	379	IH	5	1.17	√		
Niu et al. 2009; Liu et al. 2009; Niu et al. 2011	1324	2.4	379	IH	6	1.17	√		
Niu et al. 2009; Liu et al. 2009; Niu et al. 2011	1324	2.4	379	IH	7	1.17	√		
Niu et al. 2009; Liu et al. 2009; Niu et al. 2011	1324	2.4	379	IH	8	1.17	√		
Niu et al. 2009; Liu et al. 2009; Niu et al. 2011	1324	2.4	379	IH	9	1.17	√		
Niu et al. 2009; Liu et al. 2009; Niu et al. 2011	1324	2.4	379	IH	10	1.17	√		
Niu et al. 2009; Liu et al. 2009; Niu et al. 2011	1324	2.4	379	IH	11	1.17	√		
Garten et al. 2009	183	14.4	1322	OTC	1	1.20	√		

Garten et al. 2009	183	14.4	1322	OTC	2	1.20	√		
Song et al. 2019	1324	2.4	379	IH	1	0.20	√		
Song et al. 2019	1324	2.4	379	IH	2	0.20	√		
Song et al. 2019	1324	2.4	379	IH	3	0.20	√		
Song et al. 2019	1324	2.4	379	IH	4	0.20	√		
Song et al. 2019	1324	2.4	379	IH	5	0.20	√		
Ganjurjav 2017	4700	0.3	462	OTC	1	0.63	√		
Ganjurjav 2017	4700	0.3	462	OTC	1	1.21	√		
Ganjurjav 2017	4700	0.3	462	OTC	2	0.73	√		
Ganjurjav 2017	4700	0.3	462	OTC	2	2.52	√		
Ganjurjav 2017	4700	0.3	462	OTC	3	1.15	√		
Ganjurjav 2017	4700	0.3	462	OTC	3	1.73	√		
Ganjurjav 2017	4700	0.3	462	OTC	4	1.00	√		
Ganjurjav 2017	4700	0.3	462	OTC	4	1.78	√		
Ganjurjav 2017	4700	0.3	462	OTC	5	0.37	√		
Ganjurjav 2017	4700	0.3	462	OTC	5	1.00	√		
Ganjurjav 2017	4700	0.3	462	OTC	1	0.80	√		
Ganjurjav 2017	4700	0.3	462	OTC	1	1.38	√		
Ganjurjav 2017	4700	0.3	462	OTC	2	1.03	√		
Ganjurjav 2017	4700	0.3	462	OTC	2	1.56	√		
Ganjurjav 2017	4700	0.3	462	OTC	3	0.36	√		
Ganjurjav 2017	4700	0.3	462	OTC	3	0.54	√		
Ganjurjav 2017	4700	0.3	462	OTC	4	1.03	√		
Ganjurjav 2017	4700	0.3	462	OTC	4	1.65	√		
Mao 2016	4003	-3.3	420	OTC	1	1.29	√		
Chen 2015	3140	0.8	398	OTC	1	1.12	√		

Chen 2015	3140	0.8	398	OTC	2	1.60	√	√	√
Chen 2015	3140	0.8	398	OTC	3	1.55	√	√	√
Tian 2014	3200	-1.7	582	OTC	1	0.48	√		
Tian 2014	3200	-1.7	582	OTC	1	0.60	√		
Tian 2014	3200	-1.7	582	OTC	1	0.87	√		
Tian 2014	3200	-1.7	582	OTC	1	1.29	√		
Yu et al. 2019	5305	1.3	477	OTC	1	1.02	√		
Yu et al. 2019	5305	1.3	477	OTC	1	2.69	√		
Yu et al. 2019	5305	1.3	477	OTC	2	1.04	√		
Yu et al. 2019	5305	1.3	477	OTC	2	3.49	√		
Yu et al. 2019	5305	1.3	477	OTC	3	1.12	√		
Yu et al. 2019	5305	1.3	477	OTC	3	2.59	√		
Yu et al. 2019	5305	1.3	477	OTC	4	1.55	√		
Yu et al. 2019	5305	1.3	477	OTC	4	2.85	√		
Wan et al. 2007	183	14.4	1322	OTC	1	1.45	√		
Zhou et al. 2006	345	16.2	905	IH	1	1.20	√		
Zhou et al. 2006	345	16.2	905	IH	1	3.05	√		
Saleska et al. 2002	2920	-1.2	750	IH	1	0.90	√		
Saleska et al. 2002	2920	-1.2	750	IH	2	0.90	√		
Xu et al. 2015	345	16.2	905	IH	1	2.10	√		
Xu et al. 2015	345	16.2	905	IH	3	1.40	√		
Xu et al. 2015	345	16.2	905	IH	4	1.40	√		
Xu et al. 2015	345	16.2	905	IH	5	1.70	√		
Xu et al. 2015	345	16.2	905	IH	6	2.10	√		
Xu et al. 2015	345	16.2	905	IH	7	2.20	√		
Xu et al. 2015	345	16.2	905	IH	8	1.40	√		

Xu et al. 2015	345	16.2	905	IH	9	1.10	√		
Xu et al. 2015	345	16.2	905	IH	10	0.60	√		
Xu et al. 2015	345	16.2	905	IH	11	1.30	√		
Xu et al. 2015	345	16.2	905	IH	12	1.30	√		
Xu et al. 2015	345	16.2	905	IH	13	1.30	√		
Sharkhuu et al. 2016	1670	-4.5	290	OTC	1	0.70	√		
Sharkhuu et al. 2016	1670	-4.5	290	OTC	1	0.52	√		
Sharkhuu et al. 2016	1670	-4.5	290	OTC	2	1.34	√		
Sharkhuu et al. 2016	1670	-4.5	290	OTC	2	0.30	√		
Sharkhuu et al. 2016	1670	-4.5	290	OTC	3	0.91	√		
Sharkhuu et al. 2016	1670	-4.5	290	OTC	3	0.70	√		
Graham et al. 2014	590	9.4	1434	Others	3	4.46	√	√	√
Wang et al. 2018; Gu et al.2015	1102	2.6	271	OTC	1	0.50	√		
Wang et al. 2018; Gu et al.2015	1102	2.6	271	OTC	2	1.30	√		
Wang et al. 2018; Gu et al.2015	1102	2.6	271	OTC	3	1.40	√		
Wang et al. 2018; Gu et al.2015	1102	2.6	271	OTC	4	0.80	√		
Wang et al. 2018; Gu et al.2015	1102	2.6	271	OTC	5	1.60	√		
Wertin et al. 2017	1227	14.4	241	IH	1	1.81	√		
Hill et al. 2015	28	7.8	1250	Others	1	3.04	√		
Zou et al. 2018	260	9.9	857	IH	1	0.33	√		
Lellei-Kovács et al. 2008	110	10.4	505	Others	1	2.21	√		
Lellei-Kovács et al. 2008	110	10.4	505	Others	2	0.36	√		
Lellei-Kovács et al. 2008	110	10.4	505	Others	3	1.85	√		
Kang et al. 2019	1456	3.4	248	IH	1	1.57	√		
Zhang & Hong 2014	1456	3.4	248	IH	1	0.50	√		
Zhang & Hong 2014	1456	3.4	248	IH	2	1.10	√		

Shan et al. 2009; Wang 2012	1456	3.4	248	IH	1	1.16	√		
Shan et al. 2009; Wang 2012	1456	3.4	248	IH	2	1.71	√		
Wang 2014	1456	3.4	248	IH	1	0.57	√		
Wang 2014	1456	3.4	248	IH	2	0.54	√		
Li 2017	992	3.6	314	IH	1	0.99	√	√	√
Li 2017	992	3.6	314	IH	2	1.12	√	√	√
Li 2017	992	3.6	314	IH	3	1.45	√	√	√
Li 2017	1393	2.2	380	IH	1	0.92	√	√	√
Li 2017	1393	2.2	380	IH	2	0.67	√	√	√
Li 2017	1393	2.2	380	IH	3	0.31	√	√	√
Li 2017	1484	-2.7	454	IH	1	0.52	√	√	√
Li 2017	1484	-2.7	454	IH	2	0.17	√	√	√
Li 2017	1484	-2.7	454	IH	3	0.60	√	√	√
Wang et al. 2019	3290	0.1	387	OTC	1	2.00	√	√	√
Wang et al. 2019	3290	0.1	387	OTC	2	2.00	√	√	√
Wang et al. 2019	3290	0.1	387	OTC	3	2.00	√	√	√
Li 2019	3290	0.08	387	OTC	1	2.00	√		
Li 2019	3290	0.08	387	OTC	2	2.00	√		
Li 2019	3290	0.08	387	OTC	3	2.00	√		
Zhao et al. 2019	4730	-0.6	415	IH	1	1.50	√	√	√
Zhao et al. 2019	4730	-0.6	415	IH	2	1.50	√	√	√
Yan 2018	3561	1.1	752	OTC	4	1.29	√		
Li et al. 2020	4500	0.3	462	OTC	1	1.70	√		
Li et al. 2020	4500	0.3	462	OTC	2	1.70	√		
Li et al. 2020	4500	0.3	462	OTC	3	1.70	√		
Li et al. 2020	4500	0.3	462	OTC	4	1.70	√		

Li et al. 2020	4500	0.3	462	OTC	5	1.70	√		
Lin et al. 2011	3200	-1.7	570	IH	1	1.45	√		
Lin et al. 2011	3200	-1.7	570	IH	2	1.45	√		
Lin et al. 2011	3200	-1.7	570	IH	3	1.45	√		
Lv et al. 2020	3200	-1.7	560	IH	8	1.60	√		
Chen et al. 2016	3140	1.3	408	OTC	1	1.03	√		
Chen et al. 2016	3140	1.3	408	OTC	2	1.03	√	√	√
Chen et al. 2016	3140	1.3	408	OTC	3	1.03	√	√	√
Zong et al. 2013	4333	1.3	477	OTC	3	1.80	√		
Peng et al. 2015	4635	-3.8	291	IH	1	1.67	√	√	√
Peng et al. 2015	4635	-3.8	291	IH	2	1.67	√	√	√
Peng et al. 2014	4635	-3.8	291	IH	2	1.59	√		
Peng et al. 2014	4635	-3.8	291	IH	3	1.59	√		
Peng et al. 2020	4635	-3.8	290	IH	8	0.60	√	√	√
Peng et al. 2016	4635	-3.8	290	IH	2	2.27	√	√	√
Xue et al. 2015	4635	-3.8	290	IH	4	2.27	√	√	√
Xiong et al. 2014	4635	-3.8	290	IH	1	1.00	√		
Xiong et al. 2014	4635	-3.8	290	IH	1	2.00	√		
Shi et al. 2012	3400	2.8	718	OTC	1	1.00	√		
Zhao et al. 2018	4950	5.8	421	Others	2	2.30	√		
Zhao et al. 2018	5200	3.9	390	Others	2	4.20	√		
Zhao et al. 2018	5200	3.9	390	Others	2	1.90	√		
Zhao et al. 2018	4950	5.8	421	Others	3	2.30	√		
Zhao et al. 2018	5200	3.9	390	Others	3	4.20	√		
Zhao et al. 2018	5200	3.9	390	Others	3	1.90	√		
Yan et al. 2021	3500	1.5	750	IH	1	1.49	√	√	√

Yan et al. 2021	3500	1.5	750	IH	1	2.38	√	√	√
Yan et al. 2021	3500	1.5	750	IH	2	1.78	√	√	√
Yan et al. 2021	3500	1.5	750	IH	2	3.19	√	√	√
Yan et al. 2021	3500	1.5	750	IH	3	1.20	√	√	√
Yan et al. 2021	3500	1.5	750	IH	3	2.18	√	√	√
Yan et al. 2021	3500	1.5	750	IH	4	1.37	√	√	√
Yan et al. 2021	3500	1.5	750	IH	4	2.48	√	√	√
Yan et al. 2021	3500	1.5	750	IH	5	1.71	√	√	√
Yan et al. 2021	3500	1.5	750	IH	5	2.67	√	√	√
Yan et al. 2022	3500	1.5	750	IH	6	1.50	√		
Yan et al. 2022	3500	1.5	750	IH	6	2.50	√		
Yu et al. 2020	1409	4.3	256	IH	4	2.60	√		
Yu et al. 2020	1409	4.3	256	IH	1	3.10	√		
Nyberg and Hovenden 2020	890	6.0	720	OTC	4	0.55	√		
Song et al. 2020	1324	2.4	382	IH	1	0.73	√		
Song et al. 2020	1324	2.4	382	IH	2	0.73	√		
Song et al. 2020	1324	2.4	382	IH	3	0.73	√		
Song et al. 2020	1324	2.4	382	IH	4	0.73	√		
Song et al. 2020	1324	2.4	382	IH	5	0.73	√		
Wang et al. 2021	3200	-1.2	426	IH	1	2.30	√		
Wang et al. 2021	3200	-1.2	426	IH	2	2.30	√		
Wang et al. 2021	3200	-1.2	426	IH	3	2.30	√		
Wang et al. 2021	3200	-1.2	426	IH	4	2.30	√	√	√
Wang et al. 2021	3200	-1.2	426	IH	5	2.30	√	√	√
Wang et al. 2021	3200	-1.2	426	IH	6	2.30	√	√	√
Wang et al. 2021	3200	-1.2	426	IH	7	2.30	√	√	√

Oliveira et al. 2020	580	21.9	1508	IH	5	1.30	√		
Tiwari et al. 2021	4000	2.9	1500	OTC	3	1.00	√		
Tiwari et al. 2021	4000	2.9	1500	OTC	3	2.19	√		
Zhang et al. 2023	500	9.0	457	OTC	1	1.76	√		
Zhang et al. 2023	500	9.0	457	OTC	1	1.76	√		
Wang et al. 2023	415	6.1	425	OTC	3	1.30	√		
Luo et al. 2023	2544	2.2	673	OTC	1	1.40	√		
Luo et al. 2023	2631	2.2	673	OTC	1	1.38	√		
Luo et al. 2023	2700	2.2	673	OTC	1	0.97	√		

Supplementary References

1. Nottingham, A. T., Meir, P., Velasquez, E. & Turner, B. L. Soil carbon loss by experimental warming in a tropical forest. *Nature* **584**, 234-237 (2020).
2. Pries, C. E. H., Castanha, C., Porras, R. C. & Torn, M. S. The whole-soil carbon flux in response to warming. *Science* **355**, 1420-1422 (2017).
3. Soong, J. L. et al. Five years of whole-soil warming led to loss of subsoil carbon stocks and increased CO_2 efflux. *Sci. Adv.* **7**, eabd1343 (2021).
4. Hanson, P. J. et al. Rapid net carbon loss from a whole-ecosystem warmed peatland. *AGU Adv.* **1**, e2020AV000163 (2020).
