# Supplementary material for: Whole-soil warming leads to substantial soil carbon emission in an alpine grassland
Source: Nat Commun. 2024 May 27;15:4489. doi: 10.1038/s41467-024-48736-w (PMC11130387; doi:10.1038/s41467-024-48736-w)
Supplement: Supplementary file 3 — Reporting Summary [file 41467_2024_48736_MOESM3_ESM.pdf]

nature portfolio

Corresponding author(s): Biao Zhu
Last updated by author(s): May 3, 2024

Reporting Summary

Nature Portfolio wishes to improve the reproducibility of the work that we publish. This form provides structure for consistency and transparency in reporting. For further information on Nature Portfolio policies, see our Editorial Policies and the Editorial Policy Checklist.

Statistics

For all statistical analyses, confirm that the following items are present in the figure legend, table legend, main text, or Methods section.

n/a	Confirmed	
☐	☒	The exact sample size (*n*) for each experimental group/condition, given as a discrete number and unit of measurement
☐	☒	A statement on whether measurements were taken from distinct samples or whether the same sample was measured repeatedly
☐	☒	The statistical test(s) used AND whether they are one- or two-sided<br>*Only common tests should be described solely by name; describe more complex techniques in the Methods section.*
☐	☒	A description of all covariates tested
☐	☒	A description of any assumptions or corrections, such as tests of normality and adjustment for multiple comparisons
☐	☒	A full description of the statistical parameters including central tendency (e.g. means) or other basic estimates (e.g. regression coefficient) AND variation (e.g. standard deviation) or associated estimates of uncertainty (e.g. confidence intervals)
☐	☒	For null hypothesis testing, the test statistic (e.g. *F*, *t*, *r*) with confidence intervals, effect sizes, degrees of freedom and *P* value noted<br>*Give P values as exact values whenever suitable.*
☒	☐	For Bayesian analysis, information on the choice of priors and Markov chain Monte Carlo settings
☒	☐	For hierarchical and complex designs, identification of the appropriate level for tests and full reporting of outcomes
☐	☒	Estimates of effect sizes (e.g. Cohen's *d*, Pearson's *r*), indicating how they were calculated

Our web collection on statistics for biologists contains articles on many of the points above.

Software and code

Policy information about availability of computer code

Data collection	n/a
Data analysis	Open source code R 4.0.3 is used for statistical analyses (R core team).

For manuscripts utilizing custom algorithms or software that are central to the research but not yet described in published literature, software must be made available to editors and reviewers. We strongly encourage code deposition in a community repository (e.g. GitHub). See the Nature Portfolio guidelines for submitting code & software for further information.

Data

Policy information about availability of data

All manuscripts must include a data availability statement. This statement should provide the following information, where applicable:

- Accession codes, unique identifiers, or web links for publicly available datasets
- A description of any restrictions on data availability
- For clinical datasets or third party data, please ensure that the statement adheres to our policy

The data and code that support the findings of this study are openly available in figshare at https://doi.org/10.6084/m9.figshare.24921495.v2

Research involving human participants, their data, or biological material

Policy information about studies with human participants or human data. See also policy information about sex, gender (identity/presentation), and sexual orientation and race, ethnicity and racism.

Reporting on sex and gender	n/a
Reporting on race, ethnicity, or other socially relevant groupings	n/a
Population characteristics	n/a
Recruitment	n/a
Ethics oversight	n/a

Note that full information on the approval of the study protocol must also be provided in the manuscript.

Field-specific reporting

Please select the one below that is the best fit for your research. If you are not sure, read the appropriate sections before making your selection.

☐ Life sciences ☐ Behavioural & social sciences ☒ Ecological, evolutionary & environmental sciences

For a reference copy of the document with all sections, see nature.com/documents/nr-reporting-summary-flat.pdf

Ecological, evolutionary & environmental sciences study design

All studies must disclose on these points even when the disclosure is negative.

Study description	We mainly investigated the effect of 4-year whole-soil warming (2018-2021) on soil CO2 efflux in alpine grassland on the Tibetan Plateau based on a whole-soil warming experiment (since 2018). Moreover, we also determined the general pattern of experimental warming on CO2 efflux by a meta-analysis across global grasslands.
Research sample	Mainly, we set up this warming experiment in an alpine grassland ecosystem (alpine meadow, one of main vegetation types in this area). The area of alpine meadow accounts for more than 44% of the area of alpine grasslands, and its carbon storage accounts for larger than 56% of the carbon storage of alpine grasslands (Yang et al., 2008).
Sampling strategy	No sample-size calculation was performed. The sample size is 4.
Data collection	All data of the whole-soil warming experiment were collected from alpine meadow ecosystem.
Timing and spatial scale	Before the experimental treatments began, we had one sampling for soil CO2 efflux. After the beginning of the experiment, there were 6 sampling dates in 2018, 10 in 2019 and 2020, and 5 in 2021 (all in growing season).
Data exclusions	No data were excluded from the analyses.
Reproducibility	Two measurement replicates were employed.
Randomization	Sampling on individual plots were successfully kept randomly.
Blinding	Blinding is not relevant since we performed no artificial control in the experimental design.

Did the study involve field work? ☒ Yes ☐ No

Field work, collection and transport

Field conditions	We chose days with no rainfall event to make data collection. The temperature were reported in previous relevant publications.
Location	Haibei National Field Research Station of Alpine Grassland Ecosystem, China (37°29'-37°45' N, 101°12'-101°23' E, 3250 m a.s.l.).
Access & import/export	n/a

Disturbance	We made minor disturbance during soil collection by using small soil core and reasonable number of soil cores sampling.

Reporting for specific materials, systems and methods

We require information from authors about some types of materials, experimental systems and methods used in many studies. Here, indicate whether each material, system or method listed is relevant to your study. If you are not sure if a list item applies to your research, read the appropriate section before selecting a response.

Materials & experimental systems

n/a	Involved in the study
☒	☐ Antibodies
☒	☐ Eukaryotic cell lines
☒	☐ Palaeontology and archaeology
☒	☐ Animals and other organisms
☒	☐ Clinical data
☒	☐ Dual use research of concern
☒	☐ Plants

Methods

n/a	Involved in the study
☒	☐ ChIP-seq
☒	☐ Flow cytometry
☒	☐ MRI-based neuroimaging

Plants

Seed stocks	n/a
Novel plant genotypes	n/a
Authentication	n/a
